# Draft Genome Sequence of Multidrug-Resistant Strain *Citrobacter portucalensis* MBTC-1222, Isolated from Uziza (Piper guineense) Leaves in Nigeria

**DOI:** 10.1128/genomeA.00123-18

**Published:** 2018-03-01

**Authors:** Etinosa O. Igbinosa, Jana Rathje, Diana Habermann, Erik Brinks, Gyu-Sung Cho, Charles M.A.P. Franz

**Affiliations:** a Department of Microbiology and Biotechnology, Max Rubner-Institut, Kiel, Germany; b Department of Microbiology, Faculty of Life Sciences, University of Benin, Benin City, Nigeria

## Abstract

In this work, we report the draft whole-genome sequence of the multiply antibiotic-resistant Citrobacter portucalensis strain MBTC-1222 isolated from the uziza leafy vegetable in Nigeria. Sequence analysis showed the assembled genome size to be 4,881,935 bp, containing 4,603 protein-coding genes, 131 pseudogenes, 7 rRNAs, 74 tRNAs, and 9 noncoding RNAs (ncRNAs).

## GENOME ANNOUNCEMENT

Citrobacter portucalensis is a Gram-negative facultative anaerobic bacterium belonging to the family Enterobacteriaceae, and the type strain, Citrobacter portucalensis A60^T^, was isolated from water in Portugal (1). Members of the genus Citrobacter occur in the intestines of humans and animals, and due to consequent fecal shedding, they can also be found in varied environments, such as water, soil, and sewage. Some Citrobacter species strains have antibiotic resistance genes, i.e., the beta-lactamase gene *ampC* and quinolone resistance gene *qnrB*, on their chromosomal DNA (2, 3). The closely related species Citrobacter freundii is an emerging opportunistic pathogen and is known to cause infections involving the urinary, gastrointestinal, or respiratory tract (4). In addition, multidrug-resistant C. freundii strains have been isolated from various vegetables, such as lettuce, salad, and ready-to-eat-salad (5, 6). In this work, we studied the antibiotic resistance of C. portucalensis MBTC-1222, isolated from uziza leaves (also known as ashanti pepper [Piper guineense]), in Nigeria using whole-genome sequencing. So far, to our knowledge, a genome sequence of a highly multidrug-resistant C. portucalensis strain isolated from vegetables has not been available. It is therefore imperative to identify and characterize antimicrobial resistance genes of C. portucalensis strains associated with vegetables.

The total genomic DNA of C. portucalensis MBTC-1222 was isolated using the peqGOLD bacterial DNA kit (Peqlab, Erlangen, Germany). The sequencing library was prepared with an Illumina Nextera XT library prep kit (Illumina, San Diego, CA, USA) and run on the MiSeq platform (Illumina) with 2 × 250 paired-end reads. In total, 943,130 paired-end and 39,707 single-end sequence reads were obtained with approximately 54-fold coverage. The reads were *de novo* assembled using SPAdes version 3.11.1 (7). The draft genome assembly consisted of 49 contigs, and the *N*_50_ value was 330,308 bp. The genome size of C. portucalensis is 4,881,935 bp, with a 52.03 mol% G+C content. The genome sequence was annotated using the Rapid Annotations using Subsystems Technology (RAST) and NCBI servers (8). It contained 4,603 protein-coding sequences, 7 rRNAs, 74 tRNAs, and 9 noncoding RNAs (ncRNAs). The comparison of antibiotic resistance genes between C. portucalensis A60^T^ and C. portucalensis MBTC-1222 was confirmed using the ResFinder server (version 2.1) (9). Both C. portucalensis strains carry genes *bla*_CMY_ and *qnrB5*, which encode resistance to beta-lactam antibiotics and fluoroquinolone antibiotics, respectively. The isolate C. portucalensis MBTC-1222 contains further antibiotic resistance genes, including those for aminoglycosides (*strA* and *strB*), beta-lactams (*bla*_TEM_), chloramphenicol (*catA2*), sulfonamide (*sul2*), tetracycline (*tetA*), and trimethoprim (*dfrA14*). The nucleotide sequence similarities of all antibiotic resistance genes were higher than 95% compared to the GenBank database.

### Accession number(s).

The whole-genome shotgun project has been deposited at DDB/ENA/GenBank under the accession no. PJEP01000000.
